# Dynamics of Functional Networks for Syllable and Word-Level Processing

**DOI:** 10.1162/nol_a_00089

**Published:** 2023-03-08

**Authors:** Johanna M. Rimmele, Yue Sun, Georgios Michalareas, Oded Ghitza, David Poeppel

**Affiliations:** Departments of Neuroscience and Cognitive Neuropsychology, Max-Planck-Institute for Empirical Aesthetics, Frankfurt am Main, Germany; College of Biomedical Engineering & Hearing Research Center, Boston University, Boston, MA, USA; Department of Psychology and Center for Neural Science, New York University, New York, NY, USA; Max Planck NYU Center for Language, Music and Emotion, Frankfurt am Main, Germany; New York, NY, USA; Ernst Strüngmann Institute for Neuroscience, Frankfurt am Main, Germany

**Keywords:** speech, word, syllable transitions, frequency tagging, MEG

## Abstract

Speech comprehension requires the ability to temporally segment the acoustic input for higher-level linguistic analysis. Oscillation-based approaches suggest that low-frequency auditory cortex oscillations track syllable-sized acoustic information and therefore emphasize the relevance of syllabic-level acoustic processing for speech segmentation. How syllabic processing interacts with higher levels of speech processing, beyond segmentation, including the anatomical and neurophysiological characteristics of the networks involved, is debated. In two MEG experiments, we investigate lexical and sublexical word-level processing and the interactions with (acoustic) syllable processing using a frequency-tagging paradigm. Participants listened to disyllabic words presented at a rate of 4 syllables/s. Lexical content (native language), sublexical syllable-to-syllable transitions (foreign language), or mere syllabic information (pseudo-words) were presented. Two conjectures were evaluated: (i) syllable-to-syllable transitions contribute to word-level processing; and (ii) processing of words activates brain areas that interact with acoustic syllable processing. We show that syllable-to-syllable transition information compared to mere syllable information, activated a bilateral superior, middle temporal and inferior frontal network. Lexical content resulted, additionally, in increased neural activity. Evidence for an interaction of word- and acoustic syllable-level processing was inconclusive. Decreases in syllable tracking (cerebroacoustic coherence) in auditory cortex and increases in cross-frequency coupling between right superior and middle temporal and frontal areas were found when lexical content was present compared to all other conditions; however, not when conditions were compared separately. The data provide experimental insight into how subtle and sensitive syllable-to-syllable transition information for word-level processing is.

## INTRODUCTION

Oscillation-based approaches to speech comprehension posit that temporally segmenting the continuous input signal is realized through phase-alignment of low-frequency (<8 Hz; delta–theta) neuronal oscillations in auditory cortex to the slow fluctuations of the speech signal at the syllabic scale (Ahissar & Ahissar, 2005; Ghitza, 2011; Ghitza & Greenberg, 2009; Gross et al., 2013; Haegens & Zion Golumbic, 2018; Lakatos et al., 2019; Meyer et al., 2020; Panzeri et al., 2010; Rimmele, Morillon, et al., 2018; Zion Golumbic et al., 2012). This imposes constraints (Rimmele, Morillon, et al., 2018) such that speech perception is optimal at syllabic rates that fall within the range of intrinsic auditory cortex oscillations in the delta–theta range (Keitel & Gross, 2016; Lakatos et al., 2005; Lubinus et al., 2021; Teng et al., 2017). Thereby, syllabic segmentation reflects spectrotemporal acoustic processing, rather than being directly related to speech intelligibility (Daube et al., 2019; Howard & Poeppel, 2010; Rimmele et al., 2015).

For tracking and segmenting syllable-sized acoustic chunks, delta–theta neuronal oscillations seem to provide a crucial neural mechanism, but how such a mechanism interacts with word-level processing is unclear. Predictions arising from multiple linguistic processing levels, e.g., phonological or syntactic/semantic processing, have been shown to modulate lower processing levels (Altmann & Kamide, 1999; Jadoul et al., 2016; Kotz & Schmidt-Kassow, 2015; Marslen-Wilson & Tyler, 1980; Scontras et al., 2015). Furthermore, several experiments have documented neural tracking at several higher linguistic processing levels, such as words, phrases, and sentences (Ding et al., 2016; Hilton & Goldwater, 2021; Kaufeld et al., 2020; Martin & Doumas, 2017; Meyer, 2017; Molinaro & Lizarazu, 2018; Rimmele et al., 2021; Rimmele, Gross, et al., 2018). Importantly, different types of evidence point to interactions of higher level linguistic processing with syllable-level segmentation involving neuronal oscillations. The modulation of phase alignment of oscillations to speech acoustics at the syllabic rate is suggested by increased speech tracking for intelligible compared to unintelligible speech (Park et al., 2015; Peelle et al., 2013; Rimmele et al., 2015; Zion Golumbic et al., 2013). This was accompanied by increased connectivity between auditory cortex and higher-level processing areas, including frontal and motor cortex (Park et al., 2015, 2018). In spite of such evidence (see also Assaneo & Poeppel, 2018; Keitel et al., 2018; Park et al., 2015; Ten Oever & Martin, 2021), how the segmentation of acoustic-based and syllable-sized processing in auditory cortex interacts with higher-level processes merits deeper study. For example, which frequencies and brain areas are involved? It is unclear whether such interactions occur with word-level processing. Speech comprehension models have focused on mapping of acoustic–phonetic to lexical processing (e.g., Marslen-Wilson & Welsh, 1978), supported by important cognitive neuroscience evidence for the processing and encoding of phonemic level information (Di Liberto et al., 2015; Mesgarani et al., 2014). However, interactions of acoustic syllable and lexical word level processing have also been suggested by some models. For example, suprasegmental information such as stress or syllable rhythm can facilitate lexical access (Cutler, 2012; Mehler et al., 1981). In summary, puzzles remain both at the linguistic/psycholinguistic and at the neural levels. Regarding the former, at what linguistic levels (e.g., word, phrasal, or sentential) syllable segmentation processes in auditory cortex interact is unclear (Keitel et al., 2018). Furthermore, whether an interaction is due to lexical-semantic (Peelle, 2012; Peelle & Davis, 2012) or phonological processing (Di Liberto et al., 2015; Mai et al., 2016) is debated. Regarding the latter, the characteristics of the network dynamics are largely unknown.

During lexical processing, the posterior middle temporal gyrus (MTG; moderately left lateralized; Binder et al., 2009) provides a sound-to-meaning interface, mapping phonological to lexical representations (Binder et al., 2009; Gow, 2012; Hickok & Poeppel, 2004; Rodd et al., 2015). Sublexical contingencies, such as syllable-to-syllable transitions (i.e., syllable-to-syllable transitions are here defined as sublexical syllabic features that have a higher within than between word probability and thus allow the “grouping” of syllables into words), contribute to word processing and have been shown to activate parts of the superior temporal sulcus (STS; Mesgarani et al., 2014; Okada & Hickok, 2006) and a dorsal-path network (Hickok & Poeppel, 2007). Additionally, parts of the inferior frontal gyrus (IFG) have been suggested to contribute to word-level processing, for example, in sublexical processing tasks reflecting sensory-motor integration (Burton et al., 2000; Moineau et al., 2005; Möttönen & Watkins, 2009) or in tasks that elicit lexical competition (Kan et al., 2006; Rodd et al., 2015; Thompson-Schill et al., 1997); whereas it has been argued that the recruitment of frontal motor areas reflects working memory processes rather than speech comprehension per se (Rogalsky et al., 2022).

We investigated neural tracking of word-level syllable-to-syllable transitions separately from lexical processing by using a foreign language, as we assume that sublexical contingencies of the foreign language are rapidly learned. Research on artificial language learning reports rapid neural tracking at the word-level aligning with behavioral learning responses (Aslin & Newport, 2012, 2014; Batterink & Paller, 2017; Buiatti et al., 2009; Chen et al., 2020; Getz et al., 2018; Henin et al., 2021; Pinto et al., 2022; Saffran et al., 1996). Neuronal tracking of learned artificial words has been shown to emerge after 9 min (Buiatti et al., 2009), and even with a block of about 3 min (Pinto et al., 2022), whereas others report rapid learning of phrasal structure after 4 min (Getz et al., 2018). Findings suggest that neuronal tracking emerges as soon as linguistic units can be identified within continuous speech. Typically, statistical learning is investigated using artificial languages. However, neural tracking at the word level likewise occurs using natural language (Lu et al., 2021; C. Luo & Ding, 2020; Makov et al., 2017; Niesen et al., 2020; Xu et al., 2022). If an individual was familiar with a language, no learning effects were reported. Interestingly, a recent study investigated neural tracking in a natural foreign language (Makov et al., 2017). The study could not distinguish syllable- and word-level tracking, as monosyllabic words were used in the foreign language condition. In the current study, we investigated neural tracking in a foreign natural language using bisyllabic words. Therefore, the tracking rate of sublexical syllable-to-syllable transitions is distinct from that of syllable processing, which allowed us to investigate whether syllable-to-syllable transitions are rapidly learned and used to identify word boundaries resulting in neural tracking at the word-level even in a foreign language.

Using a frequency-tagging paradigm (Buiatti et al., 2009; Ding et al., 2016; Ding et al., 2018; Kösem et al., 2016; Makov et al., 2017) to “align” the neuronal processing of syllables and words, we addressed the following questions: First, do lexical and syllable-transition cues of words activate a network which is left lateralized for lexical processing? And, second, do (acoustic) syllable processing in auditory cortex and word-level processing interact? In two MEG experiments, native German speakers listened to isochronously presented syllables of a native and a foreign language, presented at 4 syllables/s, resulting in a rate of 2 Hz for “disyllabic units,” that is, words or pseudo-words. The effects of lexical information (native vs. foreign condition), lexical plus syllable-to-syllable transition information (native vs. foreign pseudo-words), and syllable transition information alone (foreign vs. foreign pseudo-words) and the interaction of these processes with acoustic syllable processing are characterized.

## MATERIALS AND METHODS

### Participants

German native speakers with no previous knowledge of Turkish (self-report; note that we cannot exclude previous exposure to the sound of Turkish) participated in the two MEG experiments. In the first experiment the data of 18 (mean age: 24.32 years; *SD*: 3.3 years; female: 10) healthy right handed (Oldfield mean score: 91.38, *SD*: 15.44) participants are included in the analysis (i.e., number of participants after exclusion). In the second experiment the data of 19 (mean age: 24.46 years; *SD*: 3.73 years; female: 10) healthy right handed (Oldfield mean score: 92.74, *SD*: 14.66) new participants (with no overlap in participants across experiments) are included in the analysis. Several participants were excluded prior to the analyses, because of outlier behavioral performance (accuracy < mean −2 * *SD*; Exp. 1: *n* = 2; Exp. 2: *n* = 1) and because of technical issues (no triggers, audio problems; Exp. 1: *n* = 2; Exp. 2: *n* = 1). The study was approved by the local ethics committee of the University Hospital of the Goethe-University Frankfurt. All participants gave written informed consent for participating in the study and received monetary compensation.

### Experimental Design

#### Paradigm

**Figure 1.**
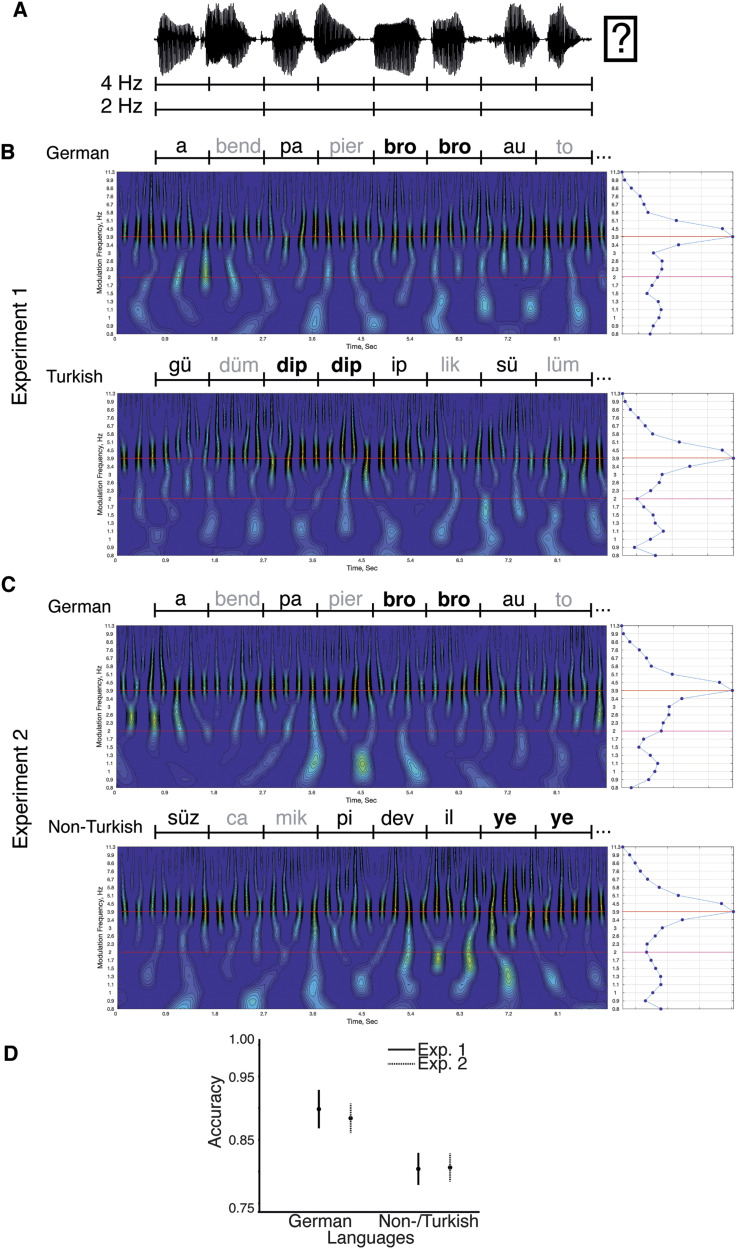
Schematic of the paradigm and acoustic material. A. The structure of a trial: Participants indicated after listening to a syllable sequence whether or not a target was present. In both experiments syllables were presented at a rate of 4 Hz (250 ms inter-onset-interval). Disyllabic word-units were presented at a rate of 2 Hz. B–C. Each panel shows the cochlear modulation spectra (Jepsen et al., 2008) averaged across cochlea channels and trials of the condition. Abscissa represents time and the ordinate represents the modulation frequencies. On the right is the modulation spectrum averaged across time. Black: first syllable; gray: second syllable. Targets were syllable repetitions (bold). B. Experiment 1: In the German condition syllables can be grouped into disyllabic words at a rate of 2 Hz. Modulation spectrum shows pronounced modulations at 4 Hz while modulations at 2 Hz are not clearly distinguishable from the activity at other frequencies. In the Turkish condition the syllables presented at 4 Hz cannot be grouped into words based on semantic knowledge, but syllable-to-syllable transition information is present. Cochlear modulation spectrum shows pronounced modulations only at 4 Hz. C. Experiment 2: The German condition corresponded to that of Experiment 1, with a different randomization of words within and across sequences; in the Non-Turkish condition, Turkish syllables were presented randomly. Thus, syllables, presented at 4 Hz, cannot be grouped into words based on semantic knowledge or syllable-to-syllable transition information. In both conditions, the cochlear modulation spectrum shows pronounced modulations at 4 Hz only. D. The performance (accuracy) did not differ across experiments. Overall the performance was higher for the German compared to the Turkish/Non-Turkish conditions (error bars: ± 1 *SEM*).

Participants were asked to listen to sequences of disyllabic words that contained either German, Turkish, or Turkish pseudo-words. In Experiment 1 (Figure 1B), sequences of German words and Turkish words were used. In Experiment 2 (Figure 1C), sequences of German words and Turkish pseudo-words (Non-Turkish) were used. Each sequence contained 38 syllables that formed 19 “disyllabic units” (words or pseudo-words). By presenting isochronous syllables, the presentation rate for syllables was fixed at 4 Hz, resulting in an occurrence of disyllabic units at 2 Hz. In order to maintain participants’ attention on the auditory stimuli, a target stimulus, which consisted of a disyllabic unit that was made of a repetition of the same syllable, was inserted in 29% of the trials (equally distributed across conditions). Participants were asked to indicate after each trial per button press whether a target stimulus was present or not (Figure 1A; index finger left and right hand, counterbalanced across participants). Each trial was followed by a jittered intertrial interval (2–3.5 s).

#### Procedure

Participants were seated in the MEG testing booth in front of a board for instructions. Stimuli were presented binaurally via insert ear-plugs (E-A-RTONE Gold 3A insert earphones; Ulrich Keller Medizin-Technik, n.d.). Participants’ responses were collected with two button boxes (Current Designs, 2022). First, participants listened to a sequence of “auditory cortex localizer sounds” and held their gaze at a fixation cross (pure tones: 0.4 s tone duration; 250 Hz and 1000 Hz, 100 repetitions, jittered intertrial interval 0.5–1.5 s). In the experiment, during each trial participants held their gaze at a fixation cross while listening to the auditory sequences. The experiment was run using Psychophysics toolbox (Brainard, 1997).

Both experiments contained 210 trials (105 per condition), grouped into 15 blocks. In total, each German and Turkish disyllabic unit was repeated 15 times. The overall duration for the experiment was 150 min, including 90 min of recording time and 60 min of preparation time, breaks, and post-recording questionnaires.

#### Stimulus selection

German disyllabic words were selected from the CELEX lexical database (Baayen et al., 1995). In order to maximize the speed of lexical access of the word as well as the predictability of the second syllable within a word, we selected words with a high frequency (CELEX spoken word frequency: MannSMln ≥ 10; CELEX zeros replaced by Leipziger Wortschatz Corpus ≥ 4000) and with transition probabilities between the two syllables (syllable transition probability; STP) ≥ 0.3%. The syllable transition probability was calculated for all disyllabic lemmas in the corpus, by dividing the wordform frequency of each lemma by the sum of the wordform frequencies of each wordform that contained the first syllable of the token (Lewis et al., 2011). Laplace transformation of zero frequencies was used (Brysbaert & Diependaele, 2012). Turkish disyllabic words were selected from the TELL database (Kuntay et al., 2009; e.g., Scharinger et al., 2011) and manually checked by a Turkish native speaker (for wordness). In total, 134 German words and 134 Turkish words were selected (noun, verb, adjectives). German and Turkish syllables were maximally matched with respect to the overall distribution of the manner of articulation for the onset phoneme of each syllable and the percentage of syllabic consonant–vowel (CV) structure.

#### Stimulus processing

In contrast to many previous studies (e.g., Buiatti et al., 2009; Ding et al., 2016) our stimuli were not based on artificial speech but on human speech recordings. Syllable stimuli produced by a female German/Turkish bilingual speaker were recorded using an external audio card (44100 Hz sampling rate). We recorded individual syllables in isolation (randomly presented to the speaker). The mean duration for German syllables was 358 ms (*SD*: 72 ms), for Turkish 334 ms (*SD*: 58 ms). Using this method, we eliminated any co-articulation and prosodic modulation between the two syllables within each word, such that acoustic cues were reduced that allow the prediction of the second syllable based on the first syllable (i.e., no acoustic cues were present at 2 Hz; Figure 1B–C). Consequently, the prediction of the second syllable in the German condition relies on higher level linguistic processing (e.g., lexical access of the word and/or sublexical syllable-to-syllable transition information; Table 1). Although the Turkish condition contains no lexical cues for grouping the syllables into words (for individuals without knowledge of Turkish), it contains sublexical syllable-to-syllable transition information with higher within word compared to between word probability (Table 1). In contrast, the Non-Turkish condition was constructed to contain no cues for word grouping.

**Table 1.** Syllable-to-syllable transitions

**Experiment 1**				
**German**				
Measurement	Within word	Between word	Difference	*p* value
Syllable identity	0.89	0.05	0.84	*p*s < 0.001
Syllable CV	0.43	0.29	0.14	*p*s < 0.001
Syllable onset	0.30	0.06	0.24	*p*s < 0.001
Initial phoneme manner	0.38	0.25	0.13	*p*s < 0.001
Rime	0.45	0.06	0.40	*p*s < 0.001
Phoneme across syllable boundary	0.28	0.07	0.21	*p*s < 0.001
**Turkish**				
Measurement	Within word	Between word	Difference	*p* value
Syllable identity	0.72	0.06	0.67	*p*s < 0.001
Syllable CV	0.50	0.37	0.13	*p*s < 0.001
Syllable onset	0.18	0.08	0.10	*p*s < 0.001
Initial phoneme manner	0.24	0.29	−0.05	*p*s < 0.001
Rime	0.38	0.06	0.33	*p*s < 0.001
Phoneme across syllable boundary	0.23	0.08	0.15	*p*s < 0.001
**Experiment 2**				
**German**				
Measurement	Within word	Between word	Difference	*p* value
Syllable identity	0.89	0.05	0.84	*p*s < 0.001
Syllable CV	0.43	0.29	0.14	*p*s < 0.001
Syllable onset	0.29	0.06	0.23	*p*s < 0.001
Initial phoneme manner	0.38	0.24	0.13	*p*s < 0.001
Rime	0.45	0.06	0.40	*p*s < 0.001
Phoneme across syllable boundary	0.28	0.07	0.21	*p*s < 0.001
**Non-Turkish**				
Measurement	Within pseudo-word	Between pseudo-word	Difference	*p* value
Syllable identity	0.07	0.06	0.007	*p*1 > 0.100
				*p*2 < 0.001
				*p*3 = 0.064
Syllable CV	0.43	0.43	<0.001	*p*s > 0.1
Syllable onset	0.08	0.08	0.001	*p*s > 0.1
Initial phoneme manner	0.25	0.25	−0.006	*p*s > 0.1
Rime	0.06	0.06	0.004	*p*1 > 0.1
				*p*2 = 0.074
				*p*3 > 0.100
Phoneme across syllable boundary	0.08	0.07	0.001	*p*s > 0.100

*Note*. For each measurement, average transitional probabilities between consecutive syllables within word boundary (within word/pseudo-word) and across word boundary (between word/pseudo-word), the average difference between those measures, and the *p* value (Mann–Whitney–Wilcoxon tests) are displayed for each experiment and the German and Turkish/Non-Turkish conditions. Note that transition probabilities and differences are displayed as averaged over the three different stimulus sets, and *p* values are further differentiated in case different results were observed for the sets. *P* values are displayed corrected for multiple comparison using Bonferroni correction. CV: consonant–vowel.

Using Praat Vocal Toolkit (Boersma, 2001; Corretge, 2022), the selected syllables tokens were high-pass filtered at 60 Hz, compressed in duration (250 ms; note that syllables starting with a plosive consonant were compressed to 230 ms and a 20 ms silence period was added in the beginning to simulate the oral occlusion before the burst of plosive consonants), and normalized in peak-amplitude and pitch contour (at 250 Hz). Overall, three different sets of stimulus sequences (used for different participants) were created for each condition, in the following way: Word stimuli were created by concatenating the two syllables of each word. For the Non-Turkish condition, disyllabic pseudo-word stimuli were created by concatenating two syllables that were quasi-randomly selected from all Turkish syllable stimuli (with an equal probability of each syllable to be at a first/second syllable position, mean probability of occurrence as first syllable for the three stimulus sets: set1 = 0.48, set2 = 0.5, set3 = 0.5). Each sequence was created by concatenating randomly selected disyllabic stimuli. For the German condition, since the word list contained several grammatical classes, we specifically checked the word order of each sequence to eliminate all possible formation of linguistic phrases as well as compound words by consecutive words. For Turkish and Non-Turkish conditions, sequences were checked to avoid “German-like” homophones.

### MRI and MEG Data Acquisition

The MRI scanning was acquired on a Siemens 3T Magnetom Trio scanner (Siemens Medical Solutions, 2022; standard 1 mm T1-weighted MPRAGE). Vitamin-E capsules were used to mark anatomical landmarks (nasion, left and right pre-auricular points). For the MEG recordings, a 269-channel whole-head system (Omega 2000; CTF MEG Neuro Innovations, 2021) situated in a magnetically shielded room was used. Data recording was performed with a sampling rate of 1200 Hz, an online low pass filtered (cut-off: 300 Hz) and online denoising (higher-order gradiometer balancing). The head position relative to the MEG sensors was continuously tracked and head displacement was corrected in the breaks using the Fieldtrip toolbox (Oostenveld, et al., 2011; Stolk et al., 2013).

## RESULTS

### Statistical Analysis

#### Syllable-to-syllable transition probability analysis

Syllable transition probabilities present in the stimulus sequences between and within words (and pseudo-words) were computed for all conditions (German, Turkish, Non-Turkish) and separately for the three stimulus sets (i.e., that were used for different participants; see Table 1). Average syllable transition probabilities between consecutive syllables within word boundary (within word) and across word boundary (between word) in German, Turkish, and Non-Turkish sequences were computed for the following phonological measurements: syllable identity, syllable CV pattern (syllable CV), syllable onset phoneme (onset), initial phoneme manner of articulation, rime (corresponding to a sub-syllabic unit that groups the vowel nucleus and the coda consonant(s) of a syllable), and phonemes across syllable boundary. Syllable transition probabilities were computed following the classical definition of transitional probabilities (also termed “conditional probabilities”) between two elements (Saffran et al., 1996). Accordingly, the transitional probability of Syllable 2 (Syl2) given Syllable 1 (Syl1) was computed as follows (with frequencies computed based on occurrence in the CELEX corpus):

$$\frac{\text{Frequency}\;\text{of}\;\text{the}\;\text{pair}\;Syl1-Syl2}{\text{Frequency}\;\text{of}\;Syl1}$$

Mann-Whitney-Wilcoxon tests were conducted separately for each stimulus set, condition, and experiment in order to test whether the transitional probabilities between syllables are significantly higher within word than between word. *P* values were corrected for multiple comparison using Bonferroni correction.

#### MRI data analysis

For MRI and MEG data analyses, we used the FieldTrip toolbox (https://fieldtrip.fcdonders.nl) (Oostenveld et al., 2011).

From the individual MRIs of all participants, probabilistic tissue maps (including cerebrospinal fluid white and gray matter) were retrieved. MRI scans were conducted for all participants, except for some participants who either did not match the MRI criteria or did not show up to the MRI scan session (Exp. 1: *n* = 5; Exp. 2: *n* = 3). In any case in which an individual MRI was missing, the standard Montreal Neurological Institute (MNI) template brain was used. In a next step, the physical relation between sensors and sources was obtained using a single shell volume conduction model (Nolte, 2003). The linear warp transformation was computed between the individual T1 MRI and the MNI template T1. The inverse of that transformation was computed, that is, a template 8 mm grid defined on the MNI template T1 was inversely transformed so that it was warped on the individual head space, based on the individual MRI and the location of the coils during the MEG recording. A leadfield (forward model) was calculated based on the warped MNI grid and the probabilistic tissue map, and used for source reconstruction. This allowed computing statistics across subjects in the MNI space with the grids of all subjects being aligned to each other.

#### MEG data analysis

##### Preprocessing.

For preprocessing, the data were band-pass filtered off-line (1–160 Hz, Butterworth filter; filter order 4) and line-noise was removed using bandstop filters (49.5–50.5, 99.5–100.5, 149.5–150.5 Hz, two-pass; filter order 4). In a common semiautomatic artifact detection procedure (i.e., the output of the automatic detection was monitored), the signal was filtered in a frequency range that typically contains muscular artifacts (band-pass: 110–140 Hz) or jump artifacts (median filter) and *z*-normalized per time point and sensor. To accumulate evidence for artifacts that typically occur in more than one sensor, the *z*-scores were averaged over sensors. We excluded trials exceeding a predefined *z*-value (muscular artifacts, *z* = 15; jumps, *z* = 30). Slow artifacts were removed by rejecting trials in which the range (min–max difference) in any channel exceeded a threshold (threshold = 0.75e−5). The data were down-sampled to 500 Hz. And epoched (−2.1–9.6 s). Trials with head movements that exceeded a threshold (5 mm) were rejected. Afterward, the different blocks of recorded MEG data were concatenated. (Note that for each block, during the recording, the head position was adjusted to the initial position of the first block). Sensors with high variance were rejected.

Eye-blink, eye-movement and heartbeat-related artifacts were removed, using independent component analysis (infomax algorithm; Makeig et al., 1996). Components were first reduced to 64 components using principal component analysis. Only in the case of a conclusive conjunction of component topography, time course, and variance across trials components were rejected. For the sensor space analysis, spherical spline interpolation was used to interpolate the missing sensors (Perrin et al., 1989).

Trials with correct responses were selected and the trial number was matched between the conditions by randomly selecting trials of the condition with fewer trials (trial number, Exp. 1: mean = 73.22, *SD* = 11.02; Exp. 2: mean = 68.68, *SD* = 10.27).

For display purposes and for the additional control analyses of statistical learning, the individual “M100 sensors” were computed based on the auditory cortex sound localizer MEG data (for details see Supporting Information, available at https://doi.org/10.1162/nol_a_00089).

##### Power.

Neuronal power was analyzed (in sensor and source space) to investigate the brain areas recruited for the processing of lexical- versus syllable-transition cues of words (2 Hz), and syllables in these conditions (4 Hz). For the sensor space analysis, the data were interpolated toward a standard gradiometer location based on the headmodel. It was epoched using a time window of 0.5–9.5 s (0–0.5 s after stimulus onset were excluded to avoid onset-related contamination) and averaged across all trials of a condition. Evoked power was computed using singletaper frequency transformation (1–7 Hz) separately for each participant of the two experiments at each condition (frequency resolution: 0.1111 Hz). At each frequency the power was contrasted by the neighboring frequency bins (± 2–3 bins). Cluster-based permutation tests using Monte Carlo estimation (Maris & Oostenveld, 2007) were performed to analyze differences between the conditions within each experiment (German vs. Turkish/Non-Turkish; dependent-sample *T* statistics) and across experiments (German vs. German and Turkish vs. Non-Turkish; independent-sample *T* statistics) at 2 Hz and 4 Hz, with an iteration of the condition affiliation (1,000 random permutations). In each permutation the cluster across sensors with the highest summed *t* value was identified by keeping only the sensors for which the difference between randomized conditions was significant at *p* = 0.05 (cluster alpha; minimum number of neighborhood sensors = 2). This resulted in a distribution of 1,000 random permutation *t* values of maximum random clusters. Then, all the identified clusters from the comparison between the actual conditions were compared to this random permutation distribution, and all the clusters with *t* value higher than the 97.5% or lower than the 2.5% of the permutation distribution were flagged as significant.

In order to analyze the brain areas recruited during the processing of lexical versus syllable-to-syllable transition cues of words (2 Hz) and syllables (4 Hz), dynamic imaging of coherent sources (DICS) was used to localize neuronal power (Gross et al., 2001). First, based on the individual leadfields a common source filter (1.333–4.666 Hz) was computed across conditions for each participant (lambda = 10%; 0.8 cm grid; note that we explored different lambda values. See Figure S1 in the Supporting Information for an analysis with lambda = 100%, which shows similar, however, slightly less conservative findings.). Second, based on the filter and Fourier transformed data (multi-taper frequency transformation; 0.1111 Hz resolution) the power at 2 Hz and 4 Hz was localized and contrasted with the neighboring frequency bins (± 2–3 bins). Differences in source power at 2 Hz and 4 Hz were tested using cluster-based permutation tests (1,000 iterations; two-sided) to analyze differences between the conditions within each experiment (German vs. Turkish and German vs. Non-Turkish; dependent-sample *T* statistics) and across experiments (German vs. German and Turkish vs. Non-Turkish; independent-sample *T* statistics) with an iteration of the condition affiliation. In each permutation the cluster across voxels with the highest summed *t* value was identified by keeping only the voxels for which the difference between randomized conditions was significant at *p* = 0.05 (cluster alpha). This resulted in a distribution of 1,000 random permutation *t* values of maximum random clusters. Then, all the identified clusters from the comparison between the actual conditions were compared to this random permutation distribution, and all the clusters with *t* value higher than the 97.5% or lower than the 2.5% of the permutation distribution were flagged as significant.

Furthermore in an additional analysis, the Brainetome atlas (Fan et al., 2016) was used to define regions of interest (ROIs; left and right superior temporal gyrus (STG), or STG1: A41_42_L/R and STG2: TE1.0_TE1.2_L/R; MTG: anterior STS; superior middle gyrus (SMG): IPL A40rv; IFG: A44v; precentral gyrus (PCG): A6cdl) to further test the condition differences at 2 Hz revealed in the cluster-test analysis. Differences between conditions at each ROI were tested separately for the hemispheres and the comparisons within each experiment (German vs. Turkish and German vs. Non-Turkish; Wilcoxon signed-rank tests) and across experiments (German vs. German and Turkish vs. Non-Turkish; Mann-Whitney-Wilcoxon tests). Bonferroni correction across ROIs and hemispheres was applied to correct for inflated *p* values.

##### Cerebroacoustic coherence.

In order to analyze the interaction between the 2 Hz word-level and the 4 Hz syllable-level processing, syllable tracking (cerebroacoustic coherence in the auditory cortex ROI at 4 Hz) was compared between conditions with or without word-level information in both experiments. Note that cerebroacoustic coherence is typically computed at the syllabic level, as this is where the most acoustic energy is contained in the speech envelope (see Figure 1C and D; Peelle et al., 2013). Therefore, first, the speech envelope was computed separately for each sentence. The acoustic waveforms were filtered in 8 frequency bands that are equidistant on the cochlear map (between 100 and 8000 Hz; third-order Butterworth filter; forward and reverse; Smith et al., 2002). The speech envelope was computed by averaging the magnitude of the Hilbert transformed signal of the 8 frequency bands separately for each sentence. The envelope was resampled to 500 Hz to match the MEG data sampling rate. Second, after the spectral complex coefficients at 4 Hz were computed for the speech envelope of each trial and the neuronal data (0.1111 Hz resolution), coherence (Rosenberg et al., 1989) between all sensors and the speech envelope was computed. A common filter (DICS; lambda = 10%; 0.8 cm grid) was multiplied with the coherence, and Fisher *z*-transformation was applied. The cerebro-acoustic coherence was averaged across voxels of the auditory cortex ROIs (STG1 and STG2) separately for the left and right hemisphere. A mixed model analysis of variance (ANOVA) was conducted to test the between-subject effect of experiment and the within-subject effects of condition (German, Turkish/Non-Turkish) and hemisphere (left, right).

##### Cross-frequency coupling.

In order to test the connectivity between auditory cortex and other brain areas, the interactions between word- and syllable-level processing, revealed by the analysis of cerebro-acoustic coherence, were further investigated by comparing cross-frequency coupling in conditions with and without lexical content and syllable transition information. Additionally, condition contrasts were tested merged across experiments (i.e., the German conditions were merged, as were the Turkish and Non-Turkish condition). Cross-frequency coupling was computed separately between the 4 Hz power envelope in a left or right auditory cortex ROI and the 2 Hz power envelope measured across the whole cortex. After trials were downsampled (100 Hz) and filtered (Butterworth, fourth order, bandpass: 1.5–2.5 Hz and 3.5–4.5 Hz), the Hilbert transform was used to compute the complex spectral coefficients at 2 Hz and at 4 Hz separately for each trial, hemisphere, condition, and participant. A common filter (across conditions and frequencies: 1.5–4.5 Hz; linearly constrained minimum variance; lambda = 10%; 0.8 cm grid) was computed and used to project each trial in source space. Power envelopes were copula normalized (Ince et al., 2017). Mutual information (MI) was estimated (Ince et al., 2017) between the 4 Hz power envelopes (at voxels of a left and right auditory cortex ROI) and 2 Hz power envelopes (measured across the whole cortex). For this analysis trials were concatenated separately for each participant and condition and MI was computed on the concatenated trials. MI was averaged across the voxels of the left and right auditory cortex ROIs, respectively. Note that no correction of multiple comparisons across permutation tests was applied.

#### Statistical learning analysis

In order to access the dynamics of power changes across the experiment (i.e., to test statistical learning across blocks; note that each block had a duration of 2.9 min with 1.45 min per condition), sensor space power was computed trial-wise by using a jack-knifing procedure (i.e., the frequency analysis was performed across *n*-block-trials-leave-one-out) and averaged across the individual M100 sensors. Otherwise the power analysis was matched to the other analyses (i.e., computed for trials with correct responses; the neural power was contrasted with the neighboring frequency bins). A linear mixed-effects model (LMM; using R Core Team, 2022, and lme4 package, Bates et al., 2015) analysis was used to test effects of experimental block order (fixed effect: block order, random effects: participant ID; in an additional model the random slope effect of a polynomial model of block order was added) on the neural power observed at 2 Hz in the Turkish condition in Experiment 1. Statistical learning would be indicated by a linear increase of neural power across blocks (polynomial first order model). Additionally to learning, a fatigue effect might occur at the end of the experiment (polynomial second order model). Models with/without a random slope effect of block order, and first and second order polynomial models were compared based on the Bayesian information criterion (BIC).

### Behavioral Measures

A mixed ANOVA was used to test the effect of lexical and syllable-to-syllable transition cues on target discrimination accuracy (Figure 1D) in Experiments 1 and 2 (between-subject factor: experiment; within-subject factor: condition; equality of variances; Levene’s test: *p*s > 0.07; normality, Shapiro–Wilk test: Bonferroni, *p*_corr_ = 0.0125; *p*s ∼ 0.027, 0.005, 0.586, 0.258). Accuracy was higher in the German compared to the Turkish/Non-Turkish conditions (*F*(1, 35) = 100.759; *p* < 0.001; η^2^ = 0.365; German: 92% vs. Turkish/Non-Turkish: 83%). There was no main effect of experiment (*F*(1, 35) = 1.794; *p* = 0.189; η^2^ = 0.025) or interaction (*F*(1, 35) = 1.303; *p* = 0.261; η^2^ = 0.005). The results indicate that the presence of lexical cues (in the German conditions) facilitated performance.

### Syllable-to-Syllable Transition Probability

Mann–Whitney–Wilcoxon tests revealed in both German and Turkish conditions significantly higher within compared to between word transitional probabilities for all experiments, stimulus sets, and measurements (for statistics see Table 1). The presence of phonological patterning at the word (2 Hz) rate in the Turkish sequences, allows for temporal grouping of Turkish syllable pairs by German listeners in the absence of lexical processes. In contrast, the Non-Turkish condition showed no significant difference (with one exception) in transitional probabilities of syllables within and between pseudo-words, suggesting that no syllable transition cues for grouping syllables into words were present. In the Non-Turkish condition for all measurements, the transitional probabilities within and between pseudoword syllables differed by less than 0.01 (1%). In contrast, in the Turkish condition differences in transitional probabilities ranged from 5% to 67% among different measurements. Nonetheless, in the Non-Turkish condition a significant effect of syllable identity transitions was observed for one stimulus set. (Note, however, that this contrast was not significant when outlier transitions, higher than 2.5 *SD*, were removed.)

### Lexical and Syllable-to-Syllable Transition Processing

**Figure 2.**
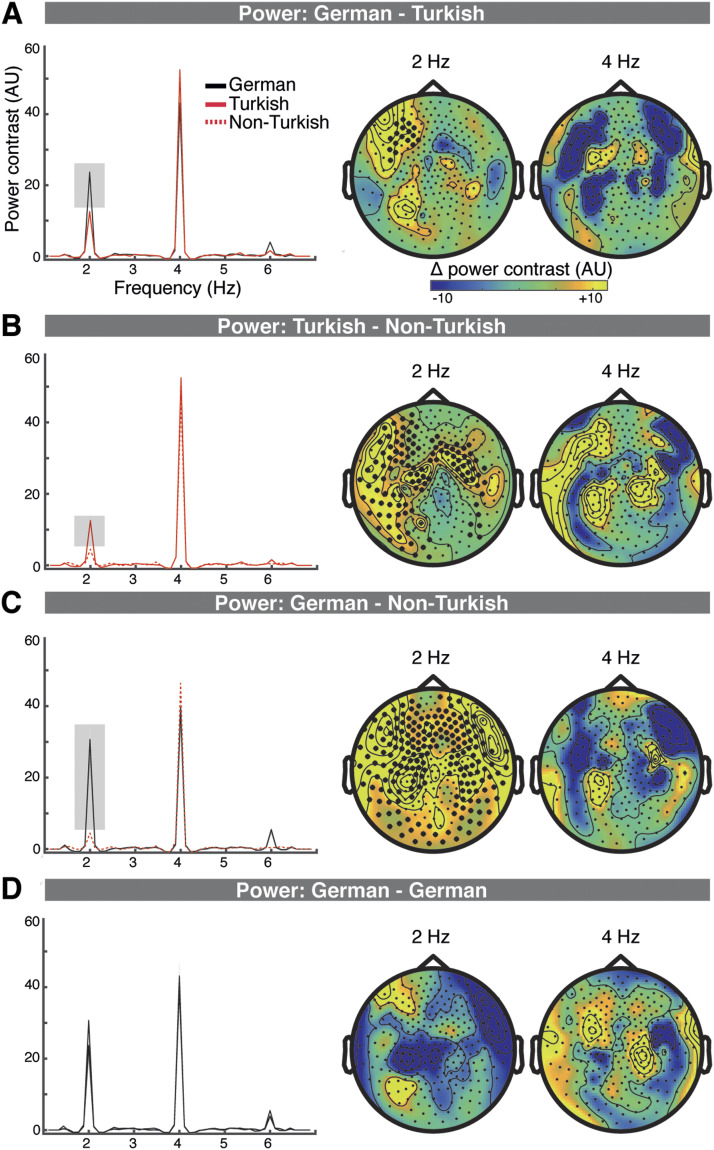
Lexical and syllable-to-syllable transition processing increases sensor space power. A–D. Power contrasts (with neighboring frequency bins) averaged across the individual M100 sensors (left); the topography of the power contrast differences between conditions at 2 Hz and 4 Hz (right). Clusters that showed significant differences are marked with black dots. A. Lexical processing, in Experiment 1, resulted in increased power at 2 Hz for the German compared to the Turkish condition at a left frontal cluster. B. Syllable transition processing, resulted in increased power in the Turkish compared to the Non-Turkish condition at 2 Hz at a left frontocentral and temporal, and a right frontocentral cluster. C. Lexical plus syllable transition processing, in Experiment 2, resulted in increased power at 2 Hz in the German compared to the Turkish condition at a broadly distributed cluster. D. No differences were detected for the across experiment comparison of the German conditions.

In the sensor-space MEG analysis, lexical access effects are reflected in Experiment 1 in power increases in the German compared to the Turkish condition at 2 Hz, at a left frontal and left temporal cluster (*p* = 0.002; Figure 2A). In source space, the comparison revealed differences at 2 Hz at a left lateralized frontal cluster (*p* = 0.022; strongest in left IFG (pars opercularis), also including left pars orbitalis, left superior frontal gyrus, right superior frontal gyrus). Non-parametric comparisons performed at 2 Hz separately for the left and right hemisphere and at the STG1, STG2, MTG, SMG, IFG, and PCG ROIs revealed no significant condition differences (left hemisphere: all *p* values > 0.0347; right hemisphere: all *p* values > 0.1119; Bonferroni corrected alpha = 0.0042).

**Figure 3.**
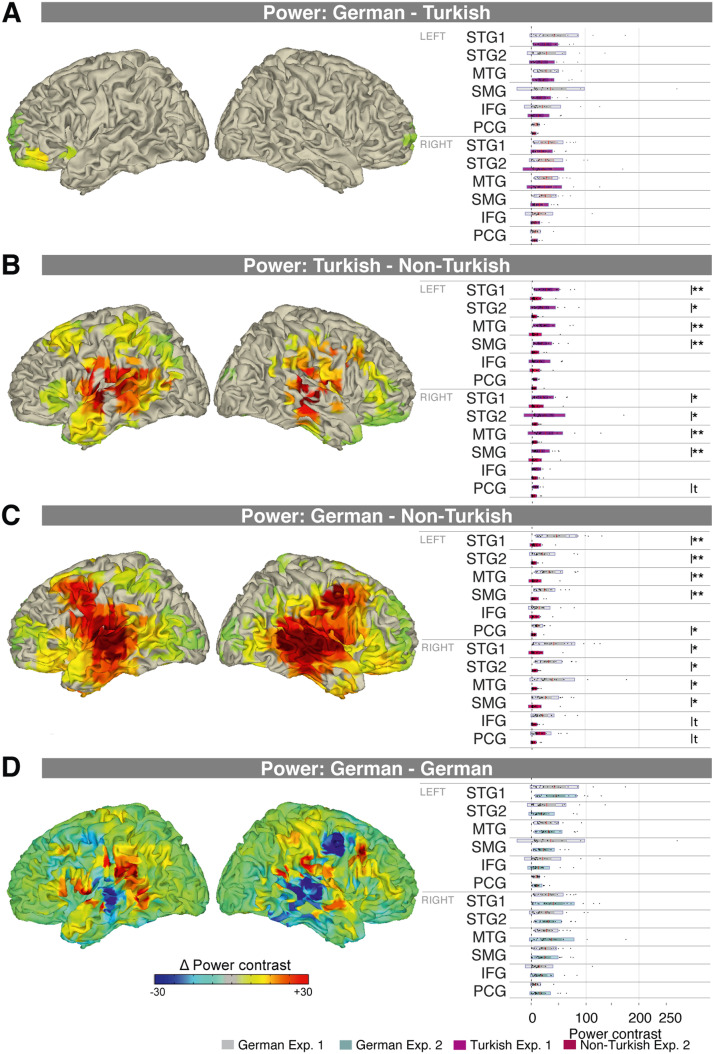
Lexical and syllable-to-syllable transition processing activates frontal and temporal cortex. A. In Experiment 1, lexical processing resulted in increased power at 2 Hz in the German compared to the Turkish condition in a cluster with stronger activations particularly in left inferior frontal brain areas (left). Exploratory comparison of condition differences at several regions of interest (ROIs; Bonferroni corrected; right). B. Syllable transition processing resulted in a broad left and a broad right hemispheric cluster showing power increases at 2 Hz (left). Condition differences were significant in several left and right hemispheric ROIs (right). C. Lexical plus syllable transition processing resulted in a broad bilateral cluster showing power increases (left). Condition differences were significant in several left and right hemispheric ROIs (right). In A–C the activity is masked by the clusters that showed significant effects. D. No significant differences were revealed in the German conditions across experiments. Note that because of the null findings, no mask was applied in this figure.

The cross-experiment comparison shows sensor-space syllable-to-syllable transition processing effects (Turkish vs. Non-Turkish) within a broad left and right hemispheric cluster (*p* = 0.002) and a broad right hemispheric cluster (*p* = 0.004; Figure 2B). In source space, syllable-to-syllable transition processing resulted in increased power in the Turkish compared to the Non-Turkish condition at a bilateral frontal, central, and temporal cluster (*p* = 0.002; with strongest activations at the STG, MTG, precentral/postcentral gyrus, and Rolandic operculum; Figure 3B). Non-parametric comparisons performed at 2 Hz revealed condition differences at the left STG1, STG2, MTG, and SMG ROIs (0.0001 < *p*s < 0.0034; Bonferroni corrected alpha = 0.0042). In the right hemisphere condition differences were significant at the STG1, STG2, MTG, and SMG ROIs (0.0006 < *p*s < 0.0037; alpha = 0.0042).

Lexical plus sublexical processing in Experiment 2 resulted in sensor power increases in the German compared to the Non-Turkish condition at a bilateral widespread cluster (*p* = 0.002; Figure 2C). In source space, connectivity was increased in the German compared to the Non-Turkish condition at a bilateral frontal, central, and temporal cluster at 2 Hz (*p* = 0.0020; with strongest activations at the STG, MTG, insula, precentral/postcentral gyrus; Figure 3C). Non-parametric comparisons performed at 2 Hz revealed significant condition differences in the left hemisphere at the STG1, STG2, MTG, SMG, and PCG ROIs (0.0001 < *p*s < 0.0015; Bonferroni corrected alpha = 0.0042). In the right hemisphere condition differences were significant at all ROIs (0.0001 < *p*s < 0.0022).

There were no significant differences detected at any cluster for the cross-experiment control comparison of the German conditions (sensor space: Figure 2D; source space: Figure 3D; the statistics can be viewed in the *t* statistic maps in Figure S3) and no condition differences at any ROI of the two hemispheres (*p*s > 0.2545). Likewise, there were no effects at 4 Hz at any comparison in sensor or source space.

### Word-Level Processing

**Figure 4.**
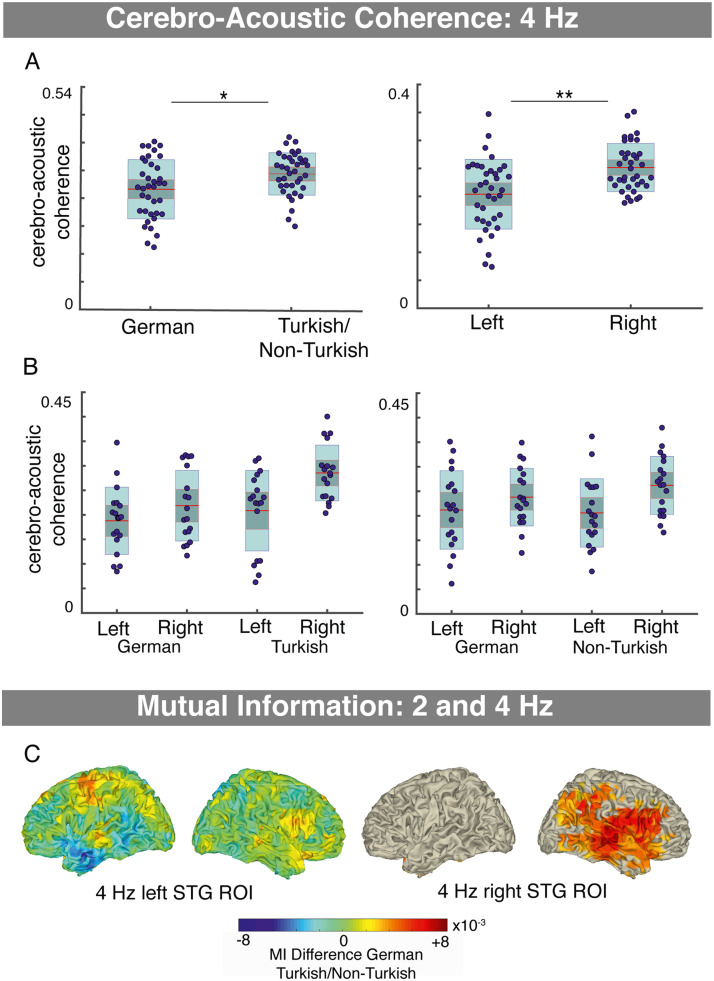
Lexical content increases interactions between word- and acoustic syllable-level processing. A. Syllable tracking in the auditory cortex ROI, measured using cerebro-acoustic coherence, was significantly reduced in conditions where lexical content was present (German conditions) compared to conditions where no lexical content was present (Turkish/Non-Turkish) (main effect condition mixed ANOVA; left column). Cerebro-acoustic coherence was significantly higher in the right compared to left hemisphere (main effect condition mixed ANOVA; right column). B. The hemispheric differences were tendency larger for the Turkish/Non-Turkish conditions, suggesting reduced speech tracking when lexical content was present particularly in the right hemisphere, and condition effects were tendency larger in Experiment 1. C. Mutual information (MI) was computed to estimate cross-frequency coupling between syllable-level processing (4 HZ) in the auditory cortex ROI and word-level processing (2 Hz), in order to further investigate the observed interaction. No cluster with significant effects was observed for the left hemispheric auditory cortex ROI. In contrast, when lexical content was present (German conditions vs. Turkish/Non-Turkish) increased MI between the right auditory cortex ROI and a cluster including inferior frontal, superior, and middle temporal, and temporal-parietal brain areas.

In order to investigate whether word-level processing affects syllable tracking in auditory cortex at 4 Hz (note that no neural power differences between conditions were observed at this frequency), a mixed ANOVA on the cerebro-acoustic coherence in the auditory cortex ROI was performed (within-subject: hemisphere, condition; between subject: experiment; equality of variances, Levene’s test: *p*s > 0.2; normality, Shapiro–Wilk test: Bonferroni, *p*_corr_ = 0.0063; *p*s ∼ 0.0565, 0.982, 0.034, 0.615, 0.052, 0.952, 0.226, 0.865). Cerebro-acoustic coherence was smaller in the German conditions of both experiments compared to the Turkish/Non-Turkish conditions (main effect of condition: (*F*(1, 35) = 7.34, *p* = 0.010, η_p_^2^ = 0.173; Figure 4A). Furthermore, there was a main effect of hemisphere (*F*(1, 35) = 12.59, *p* = 0.001, η_p_^2^ = 0.265), with overall larger cerebro-acoustic coherence in the right auditory cortex ROI (Figure 4B). There were no interaction effects (Hemisphere × Experiment: *F*(1, 35) = 2.43, *p* = 0.625, η_p_^2^ = 0.007; Hemisphere × Condition × Experiment: *F*(1, 35) = 0.155, *p* = 0.696, η_p_^2^ = 0.004). However, there was a trend for larger condition differences in Experiment 1 compared to Experiment 2 and larger hemisphere differences for the Turkish/Non-Turkish compared to the German conditions (Condition × Experiment: *F*(1, 35) = 3.188, *p* = 0.083, η_p_^2^ = 0.083; Hemisphere × Condition: *F*(1, 35) = 3.568, *p* = 0.067, η_p_^2^ = 0.093). In summary, the findings suggest when lexical content was present (i.e., in the German condition) syllable tracking in auditory cortex at 4 Hz was decreased.

To further analyze how syllable processing at 4 Hz is affected by the presence of lexical content at 2 Hz, cross-frequency coupling analyses were performed using MI. No clusters with significant effects were found for the contrasts German vs. Turkish (Exp. 1; for the left and right auditory cortex ROIs: *p*s > 0.39) or German vs. Non-Turkish (Exp. 2; for the left and right auditory cortex ROIs: *p*s = 1) or Turkish vs. Non-Turkish (across experiments; for the left and right auditory cortex ROIs: *p*s > 0.53). Additionally, the merged condition contrast was tested (German conditions were merged across experiments, and similarly Turkish/Non-Turkish conditions). For the right auditory cortex ROI, a cluster with significant differences between conditions was observed (*p* = 0.004). In the German conditions (merged across experiments), MI was increased between the 4 Hz envelope amplitude in the right auditory cortex ROI and a right hemispheric frontal, superior, middle temporal, and temporal parietal positive cluster; activity was most pronounced in the right: STG, MTG, IFG, insula, postcentral/precentral, and inferior parietal cortex (however, some activity was observed in the left PCG) compared to the conditions without lexical content (Turkish/Non-Turkish; Figure 4C). No clusters with significant condition differences were observed for the left auditory cortex ROI (*p*s > 0.39).

### Statistical Learning Analyses

A trial-wise LMM analysis was conducted on the sensor-space power at 2 Hz in the Turkish condition of Experiment 1 (Figure S4A–C). The LMM polynomial second order model shows a tendency towards a first degree effect (beta estimate: −2.82, *SE*: 1.53, *CI*: [−5.83–0.19], *t* = −1.84, *p* = 0.066) and a significant second degree effect (beta estimate: −4.06, *SE*: 1.54, *CI*: [−7.07–1.05], *t* = −2.64, *p* = 0.008; Table S1). However, if the random slope block order was added to the model first degree (beta estimate: −3.88, *SE*: 2.89, *CI*: [−9.54–1.78], *t* = −1.35, *p* = 0.178) and second degree effects were not significant (beta estimate: −4.39, *SE*: 3.16, *CI*: [−10.59–1.81], *t* = −1.39, *p* = 0.165; Table S2). The polynomial model with the random slope effect included was selected based on the BIC (BIC = 4,907; model without random slope, BIC = 4,931; polynomial first order models without/with slope had larger BIC values, BIC = 4,933 and BIC = 4,920).

## DISCUSSION

We show that the frequency-tagging paradigm can be used to distinguish aspects of lexical-level and syllable-to-syllable-transition information processing by differentiating neuronal networks activated at 2 Hz. Our findings indicate that syllable-to-syllable-transitions of a foreign language are rapidly learned and tracked, at least when there is an overlap in sublexical cues between foreign and native languages. Furthermore, we used the frequency-tagging paradigm to investigate interactions between acoustic syllable-level and word-level processing. Specifically, we found, first, decreased tracking of syllables (cerebro-acoustic coherence at 4 Hz) in auditory cortex when lexical word-level content was present compared to all other conditions; second, for the same contrast cross-frequency coupling was increased between 4 Hz activity in right auditory cortex and 2 Hz activity in a cluster that included frontal, middle, and superior temporal areas. The data might indicate interactions between lexical processing of words (here at 2 Hz) and acoustic-syllable processing (here at 4 Hz), however, further work is required. Note that at both the syllable level and the word level we are not committed to any decoding scheme. At the syllable level, we show that acoustic syllabic information—to be decoded as a whole unit or as a sequence of phonemes—is obtained within a window duration that is inside the theta range. The strongest evidence that this window is determined by theta-band oscillations comes from earlier work on the association of the drop in intelligibility of speeded speech with the upper frequency range of theta (Doelling et al., 2014; Ghitza & Greenberg, 2009). At the word level, we do not link our findings on the lexical processing to oscillations.

### Lexical and Syllable-to-Syllable Transition Processing of Words

Lexical processing, compared to sublexical syllable-to-syllable transition processing showed increased activity at a cluster of left-lateralized frontal sensors, localized to left frontal brain areas. Previous (fMRI, MEG, and lesion) research emphasized the role of the posterior middle temporal lobe in lexical-semantic processing of words, which was often reported to be left lateralized with some degree of bilateral recruitment (Figure 2A and Figure 3A; Gow, 2012; Hickok & Poeppel, 2004, 2007; Peelle, 2012; Rice et al., 2018; Thompson-Schill et al., 1997; Utman et al., 2001). Furthermore, some studies have reported a much broader network for lexical-semantic processing including the (more strongly activated) inferior frontal lobe, for example in tasks that elicit lexical competition (Kan et al., 2006; Rodd et al., 2015; Thompson-Schill et al., 1997). However, others suggested a role of the inferior frontal lobe in sublexical segmentation (Burton et al., 2000) or argued that the recruitment of frontal motor areas reflects working memory processes rather than lexical-semantic processing per se (Rogalsky et al., 2022). In light of these previous findings, our findings of increased activity in left lateralized frontal brain areas when lexical content was present need to be interpreted cautiously. Limitations of contrasting MEG source maps need to be considered, which can result in erroneous brain maps (Bourguignon et al., 2018). Given such limitations, our findings alternatively might reflect activity of sources centered in STG with slightly different center configurations in the German and Turkish conditions. For visual comparison the source maps are displayed separately per condition (Figure S3).

“Mere” syllable transition processing compared to acoustic syllable processing, in contrast, activated fronto-centro-temporal brain areas in both hemispheres (Figure 2B and Figure 3B; see also Figure 2C and Figure 3C). Previously, a functional subdivision of the temporal cortex has been proposed, with bilateral STS activations during lower-level acoustic speech processing and a left-lateralized activation of the more ventral temporal-parietal cortex during lexical-semantic processing (Binder et al., 2000, 2009). In line with this subdivision, our findings further suggest that, beyond acoustic processing, sublexical syllable-transition processing occurs bilaterally. In our paradigm, increased neuronal activity in the native language condition, which contained semantic and syllable transition cues to group syllables into words, compared to a foreign language condition, which contained only syllable transition cues (Table 1), indicates lexical processing of words. Lexical processing and syllable transition processing, however, are tightly entangled, thus an alternative possibility is that the observed increase in neuronal activity partly reflects better-learned syllable transitions in a native compared to a foreign language condition.

### Processing of Syllable-to-Syllable Transition Cues

Behavioral research suggests that sequencing of phonemes—because the distribution of phonemes varies across syllables—can be used to detect syllable-to-syllable transitions and word boundaries (Brodbeck et al., 2018; McQueen, 1998), as well as the position of syllables within words (Cutler, 2012; van der Lugt, 2001). Our findings indicate brain areas involved in using syllable transition information to process disyllabic words (Figure 2B and Figure 3B). Our findings provide evidence that even the syllable transition information present in a foreign language, that is, sublexical cues that can be used for grouping syllables into words (including phoneme transition probabilities between words) such as the onset of a syllable or the consonant-vowel pattern, which were present in both German and Turkish conditions but not the Non-Turkish condition (Table 1), can be extracted. In the present study, the stimuli were recorded and preprocessed so that acoustical cues at the word level were minimized, resulting in a prominent power peak only at the syllable rate at 4 Hz, but not at the word rate at 2 Hz (Figure 1B–C). Thus, the increased power peak at 2 Hz in the Turkish compared to the Non-Turkish condition most likely reflects the processing of syllable-transition features rather than the processing of acoustic cues. (For caveats because of acoustic cues at the word level in artificial languages, see C. Luo & Ding, 2020; Pinto et al., 2022.)

In the current study, we carefully matched the German and Turkish stimulus material with regard to the sublexical syllable-to-syllable transition cues. Possibly this enhanced the ability of participants to quickly learn and extract the sublexical contingencies of a foreign language. If the ability to extract such contingencies at the word-level depends on the similarity of these features between languages, the frequency-tagging paradigm could be used as a neurophysiological tool to investigate the phonological similarity between languages, without requiring explicit feedback from participants. In order to test statistical learning (Ota & Skarabela, 2016, 2018; Pena & Melloni, 2012; Saffran et al., 1996), we analyzed whether the tracking of sublexical syllable transitions (in the Turkish condition) varied across experimental blocks. We found a tendency toward an increase of neural power at the word level (2 Hz) across the initial experimental blocks. Furthermore, a power decrease across the last blocks was significant, indicating statistical learning and possibly fatigue related effects, respectively (Figure S4A–B). However, if the variance across participants in neural power changes across blocks was considered, these effects were not significant (Figure S4C). Visual inspection of the individual data (Figure S4C) suggests that statistical learning only occurred in some participants. Our findings are in line with previous findings that show rapid statistical learning of words and phrases in an artificial language (Buiatti et al., 2009; Getz et al., 2018; Pinto et al., 2022), with some variation in the time needed to establish word tracking (Buiatti et al., 2009, ∼9 min; Pinto et al., 2022, ∼3.22 min) or phrase tracking (Getz et al., 2018, ∼4 min). Particularly, a recent study on statistical learning in an artificial language found no effects of the block order on word-level tracking, interpreted as rapid learning within the duration of the first block (Pinto et al., 2022, 3.22 min). In line with our finding, they furthermore pointed out the high variance in whether neural tracking of words occurred at the single subject level, which was only observed in 30% of the participants.

### Interactions

Previous speech comprehension models have focused on mapping of acoustic-phonemic to lexical processing (e.g., Marslen-Wilson & Welsh, 1978). Neurophysiological data, however, provide compelling evidence for the extraction of acoustic information at the syllable level (Gross et al., 2001; H. Luo & Poeppel, 2007; Panzeri et al., 2010). What does that mean for our understanding of speech comprehension? In accordance with previous evidence, our findings show stronger syllable tracking (4 Hz; cerebro-acoustic coherence) in the right compared to the left auditory cortex (Flinker et al., 2019; Giroud et al., 2020; H. Luo & Poeppel, 2007). Crucially, syllable tracking decreased when lexical content was present (i.e., German condition; compared to when no lexical content was present), indicating an interaction between word-level and acoustic syllable-level processing. Our findings are in line with several previous findings: In frequency-tagging paradigms, lexical processing of words (in artificial word learning, or when compared with a foreign language) resulted in reduced power at the syllabic rate when words were intelligible compared to unintelligible (Buiatti et al., 2009; Makov et al., 2017; Pinto et al., 2022). In contrast, many studies have found increased syllable tracking in left auditory cortex during the processing of intelligible compared to unintelligible speech (e.g., Park et al., 2015; Peelle et al., 2013; Rimmele et al., 2015). Such controversial findings have been explained in the context of the predictive coding framework (Sohoglu & Davis, 2016). Increased intelligibility and tracking in STG due to sensory detail in the stimulus acoustic (original vs. noise-vocoded speech) was related to increased prediction errors. In contrast, increased intelligibility and reduced speech tracking in STG due to prior (linguistic) knowledge, was related to increased top-down predictions. The latter effect was observed particularly in the right hemisphere. The findings suggest that the effects of the interaction can vary depending on the paradigm, performed processes, and so on.

More specifically, in our study, acoustic syllable-level processing in right auditory cortex showed increased interactions with lexical word-level processing in right inferior frontal, superior, and middle temporal cortex (cross-frequency coupling). In line with proposals of a crucial role of the MTG as an interface between phonetic and semantic representations (Gow, 2012), our findings suggest that in addition to the inferior frontal brain areas and the STG, the MTG is involved in communicating information between syllable and word-level processing. It is likely that our findings indicate both feedforward communication from auditory cortex to higher-level processing areas and feedback from the word-level to the syllable-level. For example the first syllable might provide (temporal and/or semantic) predictions of the second syllable. Interactions between lexical and phonological processing have been shown to involve feedback from posterior MTG to posterior STG (Gow & Segawa, 2009; for review see Gow et al., 2008). Furthermore, several electrophysiological studies suggest interactions/feedback from sentential (Gow & Olson, 2016) or phrasal processing (Keitel et al., 2018), or possibly both (Park et al., 2015) to syllable processing. However, research that is particularly designed to investigate the interactions at the word level is rare (Gow & Olson, 2016; Keitel et al., 2018; Mai et al., 2016). One limitation of our findings is that effects suggesting syllable-to-word level interactions were only observed when conditions with lexical content at the word level were compared to all other conditions (Turkish/Non-Turkish), but not in separate comparisons. A possibility is that the acoustic syllable to word-level interactions were weak and the effects significant only for the larger data sets. This conjecture is in line with Pinto et al. (2022), who reported low statistic reliability of the effect of word learning on syllable tracking.

### Conclusions

Our data shed light on the contribution of syllable-to-syllable transition cues to neural processing at the word-level. Particularly, we find that sublexical syllable-to-syllable transition are rapidly tracked in a foreign language. Furthermore, the increased coupling between word- and syllable-level processing, when lexical cues are present, suggests that these processes are interactive.

## ACKNOWLEDGMENTS

We thank Marius Schneider for help with the data recording, Ilkay Isik for checking the Turkish stimulus material, Dr. Florencia Assaneo for discussions, and Dr. Klaus Frieler for statistics support.

## FUNDING INFORMATION

This work was funded by the Max Planck Institute for Empirical Aesthetics.

## AUTHOR CONTRIBUTIONS

**Johanna M. Rimmele**: Conceptualization: Equal; Formal analysis: Lead; Methodology: Lead; Project administration: Equal; Visualization: Lead; Writing – original draft: Equal; Writing – review & editing: Equal. **Yue Sun**: Conceptualization: Equal; Formal analysis: Supporting; Methodology: Supporting; Writing – review & editing: Equal. **Georgios Michalareas**: Methodology: Supporting; Visualization: Supporting; Writing – review & editing: Equal. **Oded Ghitza**: Conceptualization: Equal; Formal analysis: Supporting; Visualization: Supporting; Writing – review & editing: Equal. **David Poeppel**: Conceptualization: Equal; Funding acquisition: Equal; Methodology: Supporting; Writing – review & editing: Equal.

## DATA AVAILABILITY STATEMENT

Parts of the data are available on Edmond, the open research repository of the Max Planck Society.

## TECHNICAL TERMS

Neuronal oscillations: Oscillations that likely emerge from neuron populations, reflecting the alignment of excitability cycles and having characteristics of self-sustained oscillators and a natural frequency.
Speech tracking: The phase alignment of neuronal activity in auditory cortex (and possibly other areas) to the energy fluctuations in speech acoustics.
Sublexical: Denotes the speech processing preceding lexical access.
Syllable-to-syllable transition: Sublexical syllabic features with higher within than between word probability, allowing the grouping of syllables into words.
Frequency tagging: Approach in which linguistic (or other) units are presented isochronously to temporally align the neuronal processing of these linguistic units.
