# Supplementary material for: Dynamics of Functional Networks for Syllable and Word-Level Processing
Source: Neurobiol Lang (Camb). 2023 Mar 8;4(1):120–44. doi: 10.1162/nol_a_00089 (PMC10205074; doi:10.1162/nol_a_00089)
Supplement: Supplementary file 1 [file nol-4-1-120-s001.pdf]

Supplemental Material

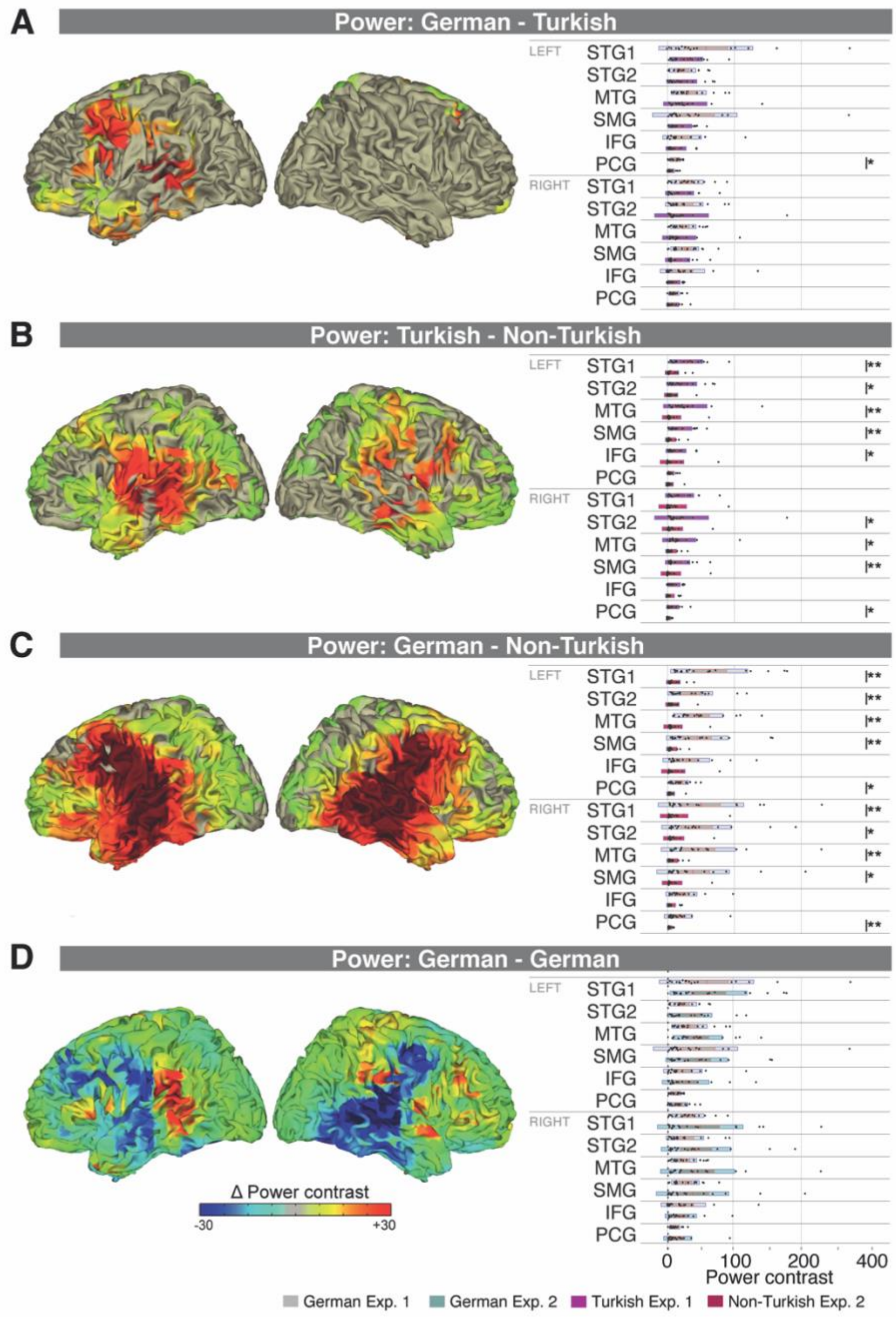

Fig. S1 Neuronal source space power (2 Hz) during lexical and syllable-to-syllable transition processing using lambda = 100%. Here we repeated the source power analysis with a different regularization parameter lambda. The analysis shows similar, however, slightly less conservative findings compared to our main analysis, where we used lambda = 10% (main manuscript: Fig. 3). Particularly, with this analysis we see a larger network that includes left middle and superior temporal cortex showing effects when lexical content was present compared to when no lexical content was present (German vs. Turkish contrast; panel A).

.

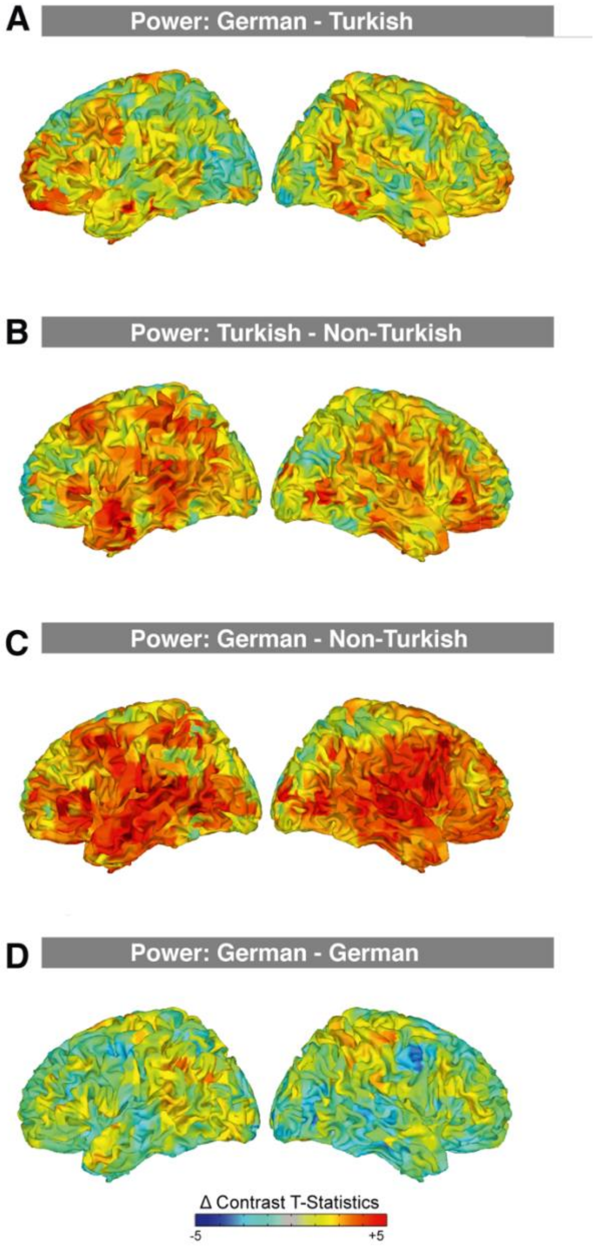

Fig. S2 Neuronal source space power (2 Hz) during lexical and syllable-to-syllable transition processing as indicated by t-statistic maps: Here the t-statistics as revealed by the cluster permutation tests on neuronal power differences between conditions (see methods section manuscript, Fig. 3) are displayed. Note that here no masks for significant effects were applied. Significant clusters were detected in all, but the control comparison (German-German) (see Fig. 3). This is indicated by high t-values across the brain areas where significant clusters were observed (A-C), and low t-values across the whole brain for the control condition (German-German) where no significant cluster was observed).

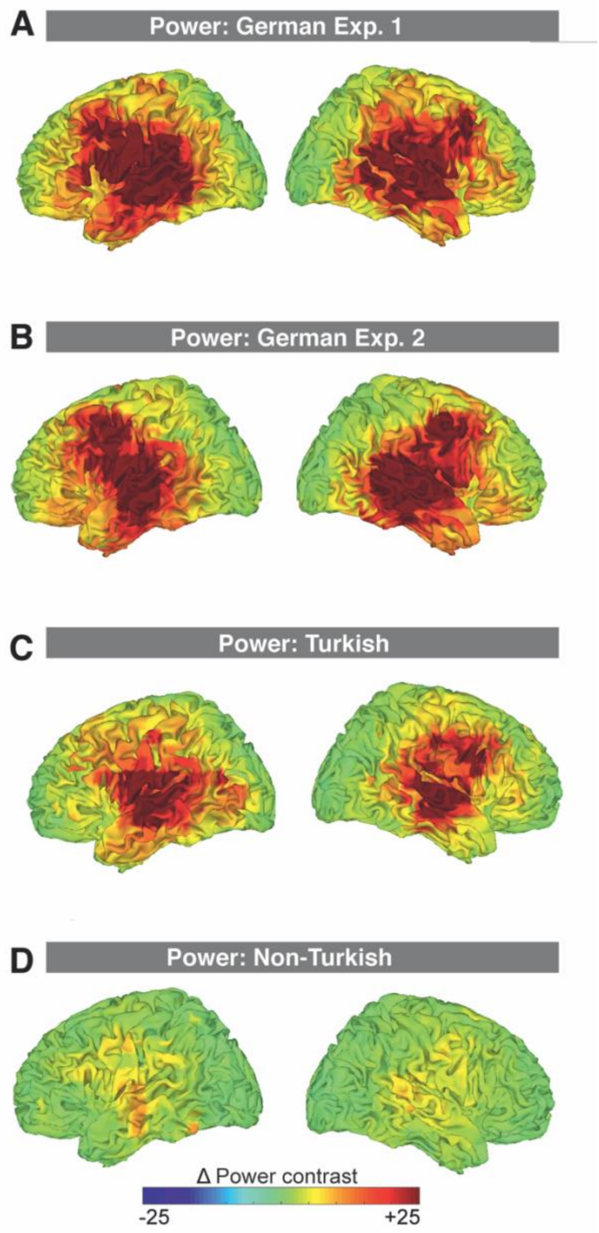

Fig. S3 Neuronal source space power (2 Hz) during lexical and syllable-to-syllable transition processing displayed separately per condition. Neural power (contrasted with the neighboring frequency bins) is displayed separately per condition (and the two experiments). The activity maps show stronger activations in inferior frontal brain areas in the German compared to the Turkish condition; however, the center of the activity seems in the superior temporal lobe.

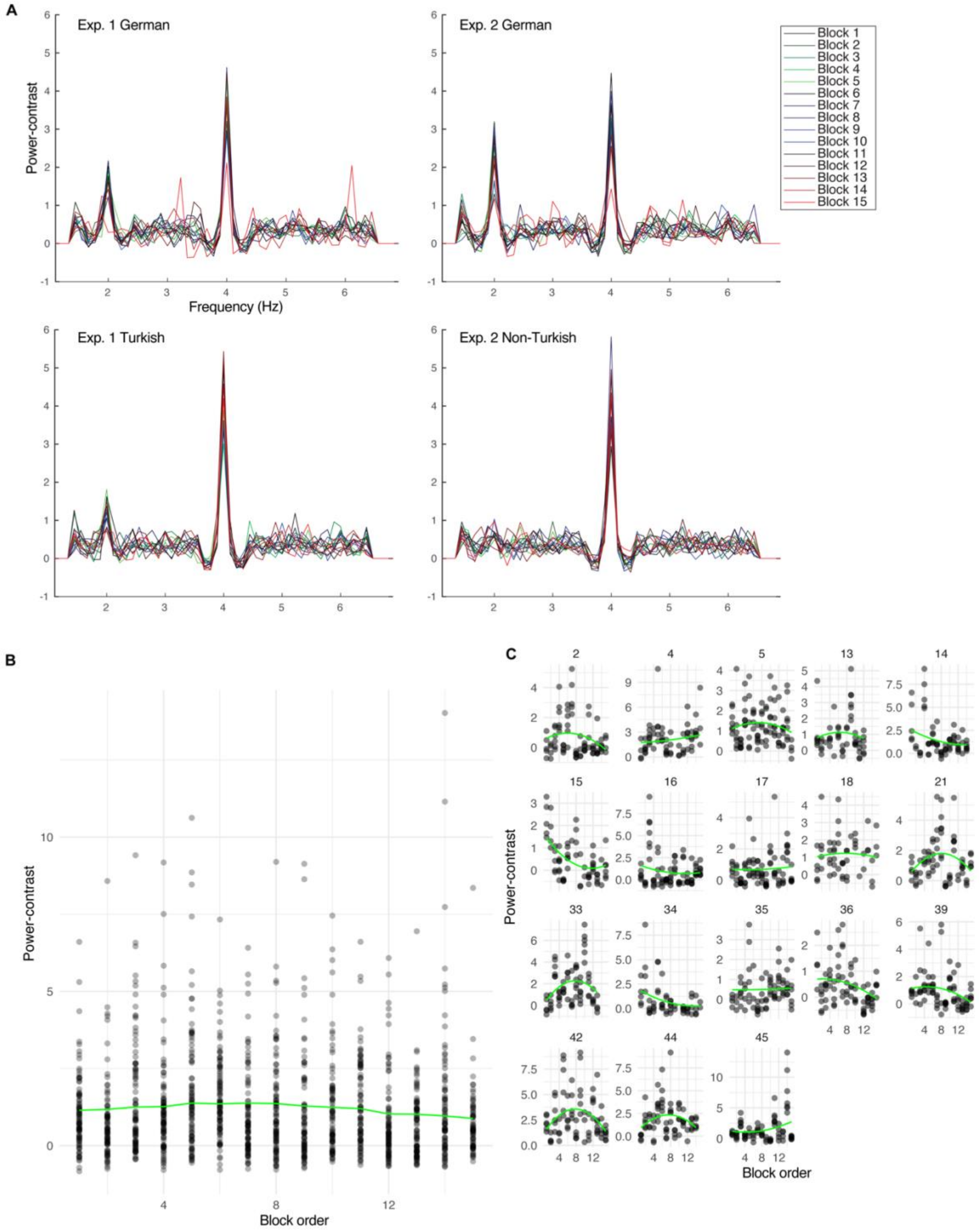

Fig. S4 Analysis of statistical learning of sublexical syllable-to-syllable contingencies in the Turkish condition at 2 Hz (Exp. 1). (A) Neural power (contrasted with the neighboring frequency bins) is displayed separately for the 15 experimental blocks (color legend) and each condition of the two experiments (German, German, Turkish, Non-Turkish). The 2 Hz and 4 Hz peak are clearly visible in all conditions, but the Non-Turkish condition, which is not showing a 2 Hz peak. Note that the signal is noisier compared to the main analysis, because neural power was computed over a few trials only (in a jack-knifing procedure trial-wise per block). Note that each block hat a duration of 2.9 min with 1.45 min per condition; (B) The 2 Hz neural power (contrasted with the neighboring frequency bins) in the Turkish condition

(Exp. 1) is displayed separately per block (block order on the x-axis), showing single trials for each participant (black dots; note that some participants had no trials for a given block, as some trials were removed because of a noisy signal in the preprocessing and only trials with correct responses were considered). A polynomial second order model was fitted to the data (green line). Note that this model did not reveal significant effects (model with random slopes of block order included). (C) The 2 Hz power data in the Turkish condition (Exp. 2) is displayed separately per block, showing all trials for each participant (participant IDs are displayed on the top of each sub-plot). A polynomial second order model was fitted to the data (green line). Visual inspection shows variance across the slope (power across blocks); with about one third of participants showing a power increase across the first blocks and a decrease across the last blocks, others showing only an increase at the beginning, only a decrease at the end, or some showing no changes with in general very low power.

Table S1 Statistical learning of sublexical contingencies was investigated using a Linear Mixed Model of neural power across blocks at 2 Hz in the Turkish condition in Exp. 1 (polynomial second order model, no random slope effect included)

	**Neural power**		
Predictors	*beta-estimates*	*CI (95%)*	*p*
(Intercept)	1.20	0.90 – 1.51	**<0.001**
block [1st degree]	-2.82	-5.83 – 0.19	0.066
block [2nd degree]	-4.06	-7.07 – -1.05	**0.008**
**Random Effects**			
σ^2	2.33		
$\tau_{00\ subject}$	0.40		
ICC	0.15		
$N_{subject}$	18		
Observations	1318		
Marginal R^2 / Conditional R^2	0.007 / 0.152		

Note that the model displayed here was not selected based on the BIC. Instead the model displayed in Table S2 was selected.

Table S2 Statistical learning of sublexical contingencies was investigated using a Linear Mixed Model of neural power across blocks at 2 Hz in the Turkish condition in Exp. 1 (polynomial second order model, with random slope effect included)

	**Neural power**		
Predictors	*beta-estimates*	*CI (95%)*	*p*
(Intercept)	1.19	0.88 – 1.49	**<0.001**

block [1st degree]	-3.88	-9.54 – 1.78	0.178
block [2nd degree]	-4.39	-10.59 – 1.81	0.165
**Random Effects**			
σ^2	2.15		
τ_{00} subject	0.41		
τ_{11} subject.poly(block, 2)1	107.24		
τ_{11} subject.poly(block, 2)2	137.72		
ρ_{01}	0.35		
	-0.46		
ICC	0.22		
N subject	18		
Observations	1318		
Marginal R^2 / Conditional R^2	0.009 / 0.224		

Note that this model was selected based on the BIC.

Supplemental Methods

M100 sensor selection. For displaying purposes and for the additional control analyses of statistical learning the individual “M100 sensors” were computed based on the auditory cortex localizer sound MEG data. These sensors reflect individual auditory cortex activity. After preprocessing, the epoched (0-0.5 sec) trials were baseline corrected (-0.4-0 sec). The M100 peak was determined for each participant, as the max. amplitude of the root mean squared data within a window of 0.07-0.160 sec. For each hemisphere the 15 sensors with max. amplitude at this time point were selected separately for each hemisphere and participant. For 5 participants no localizer MEG recordings were present (Exp. 1, n = 4) and thus the M100 sensors were selected based on the grand average of the respective experiment.
